# Go to work or report sick? A focus group study on decisions of sickness presence among offshore catering section workers

**DOI:** 10.1186/1756-0500-4-70

**Published:** 2011-03-18

**Authors:** Kariann Krohne, Liv Heide Magnussen

**Affiliations:** 1 Faculty of Health Sciences, Oslo University College, Oslo, Norway; 2 Uni Health, Bergen, Norway; 3 Department of Public Health and Primary Health Care, University of Bergen, Norway

## Abstract

**Background:**

To identify and explore the factors promoting sickness presenteeism among offshore catering section workers.

**Methods:**

Twenty men and women, working in the offshore catering section onboard three offshore oil and gas production platforms on the Norwegian Continental Shelf, participated in three focus groups. Data from the focus groups were analysed according to a phenomenological approach, and supported by theories on presenteeism.

**Results:**

The results show that the decision to attend work despite illness, first and foremost, was based on the severity of the health complaint. Other factors identified were; the individual's location once the health complaint occurred, job satisfaction, the norms of the team, and experiences of how company policies on sickness absenteeism were implemented by the catering section leaders.

**Conclusions:**

Offshore working conditions may promote sickness presenteeism. The factors promoting sickness presenteeism onboard the platforms reflected experiences of a healthy work environment.

## Background

Absence from work due to sickness has been the subject of several epidemiological studies [1-4]. In recent years, increased research attention has been paid to 'the opposite side of the coin'; sickness presenteeism [5,6]. Sickness presenteeism refers to the phenomenon of people going to work despite complaints and ill health that should prompt them to rest and take sick leave [7].

Two main research trends are recognized in this field. First and most predominantly, research that tends to view presenteeism as a negative phenomenon focuses mainly on the negative health effects of workers who attend work despite being aware that their perceived state of health justifies taking sick leave [7-11]. Associations has been shown between sickness presenteeism and sick leave [8]. Aronsson and collaborators [7] found that occupational groups who had work accumulating following a period of sickness absence reported both high sickness presenteeism and high sickness absenteeism. Increasing job stress and low income were also found to be associated with sickness absence as well as sickness presence. Economics research has also found that sickness presenteeism has negative economic consequences for companies, indicating that workplace presenteeism is a larger and more uncontrollable economic problem than sickness absence [12].

The second type of research on presenteeism focuses on the positive health aspects presenteeism may have for the individual. Work-related health-promoting factors rest on the belief that work in general is beneficial for the individual's health [13-15]. Thus, staying active and avoiding the social isolation and inactivity that often follow sickness absence will have a positive effect on the individual's health and reduce or avoid sickness absence. This is based on a biopsychosocial approach to illness or disease that focuses on the individual's ability to function within the given environment [16]. This notion is incorporated in Norwegian government policies on prevention of sickness absence [17]. In this view, presenteeism is seen as a result of a healthy workplace. A healthy workplace is considered a workplace where people are able to produce, grow, serve, and be esteemed [14,18]. Employees with high levels of job coping and job satisfaction may decide to go to work despite health complaints simply because they want to work, not because of obligation. Pride and confidence at work are previously identified core determinants of healthy working conditions [18]. In this sense, sickness presenteeism may not be a health risk factor.

Possible predictors of sickness presenteeism are divided into two main areas: work-related factors and individual factors. Work-related factors may be linked to time pressure, control regarding work tasks, relationship with colleagues, and employment conditions. Individual factors may be linked to perceived health, financial incentives, family life, and psychological factors [19], as well as attitudes from managers and fellow workers [20]. Attention should be directed towards how the individual interprets these factors when deciding whether to stay at home or go to work ill [19].

Offshore work schedules differ from most onshore work schedules, and decisions regarding going to work ill may therefore differ from the situation of onshore employees. The offshore workers are transported to the platforms by helicopter. Staying onboard, they work 12-hour shifts for 14 days, followed by a four week free period. Catering section (CS) workers largely perform strenuous and repetitive work tasks. They prepare and serve all meals and provide cleaning and laundry service.

In the present study, a qualitative method was used to gain insight into the factors affecting offshore CS workers when deciding whether going to work despite illness or health complaints.

## Methods

The focus group method was chosen for this study as it enables participants to express themselves in a flexible discussion [21], and it proves valuable in rendering in-depth information about their experiences [22]. The study was approved by the Regional Committee for Medical Research Ethics in Norway.

The focus group interviews were conducted on the Norwegian Continental Shelf in June of 2007. CS employees on three oil and gas production platforms were recruited to participate in a focus group study based on their platform's level of sickness absence. Substantial differences in sickness absence were registered between platforms. Aiming to explore if work environment influenced sickness absence, focus groups were formed on platforms with high, average and low sickness absence, respectively. The participants were recruited by the CS leaders on each platform. In total, 20 participants (12 men, eight women) formed three focus groups (group sizes six, seven, and seven). Two of the focus group sessions were conducted during working hours, and one directly after the shift ended.

In the CS, a higher number of women than men are employed. The focus group participants reported their offshore work experience to range from 2.5 years to 27 years (average 11 years). Participants' age was not registered, but our observations and their reported work experience indicated participants' age from 30 to 60 years. The average age of a CS worker, reported in a parallel questionnaire survey where 198 of 323 CS employees responded, was 46 years. Group composition reflected the occupational diversity in the CS as a whole (62% in CS service and 32% in CS kitchen) as 13 participants (65%) worked as CS service personnel (cleaning), and seven worked in the kitchen. Several focus group participants stated that, because of the company's general downsizing of onshore operations, they had accepted relocation to offshore CS even though their new offshore occupational status did not match their original profession. As an example, an onshore electrician could be working as a cleaner in the offshore CS.

The 90-minute focus group sessions were conducted in available meeting rooms aboard the platforms. In accordance with the Helsinki declaration, the moderator (KK) informed the participants about the purpose of the study, limits of confidentiality, and their right to withdraw. Informed consent was obtained from all participants. The interview was guided by eight open-ended questions on issues related to healthy work environment, management, and sickness presenteeism. Participants were encouraged to discuss these freely to enhance group interaction [23]. For the purpose of this article the main question was: *"Have you ever gone to work even if you felt you were too ill?" *The participants knew each other and a free-flowing discussion within the subject area was apparent in all three sessions. The moderator avoided influencing the discussion, and explored both the positive and negative comments made by participants. At the end of the sessions participants were invited to verify a short oral summary presented by the moderator. An assistant moderator was present and took written notes throughout the sessions. All sessions were tape-recorded and transcribed verbatim.

The analysis followed Giorgi's phenomenological method [24], as modified by Malterud [25]: To ensure transparency and reliability, the data were independently read and analyzed by both authors. Four steps were followed: (i) transcripts were read by both authors to gain a contextualized impression of the discussions, and preliminary themes discussed and chosen; (ii) units of meaning were identified and coded; (iii) the meaning in the coded groups was condensed, and; (iv) the descriptions of presenteeism-related factors were generalized. Categories organizing the main reasons for going to work when feeling ill were negotiated until agreement between the authors was achieved. General agreement between the authors was high. Analysis was data driven.

The findings are presented as descriptive summaries under five subheadings: Ill or just feeling unwell?, Location matters, Job satisfaction, Being a team worker, and Leadership and company policy. Quotes are translated from Norwegian, and coded by group and participant number.

## Results

### Ill or just feeling unwell?

All participants claimed they had gone to work despite experiencing health problems that they believed could have prompted them to rest and take sick leave. The reasons given for attending work when ill varied. However, the decision was primarily based on the question: Am I ill or just feeling unwell? Unwell was defined as feeling 'under the weather', but capable of working through the day, a few days, or even the remainder of the shift if necessary: *If you're a bit unwell or have a cold, or maybe you're feeling feverish, you'll go to work anyway (3/7)*.

However, if they felt more severely ill offshore, the participants would be ordered to stay in their cabins or be transported home. If their medical condition was contagious, their presence on board was considered a safety violation: *If you have a really bad flu that your colleagues might catch, then you stay at home! (3/4)*. Fellow workers might also pressure colleagues to stay away from work for this reason. If they became ill at home, a medical doctor was consulted to certify their absence.

### Location matters

The shift shapes the continuity of the work and controls the logistics of working offshore. If a smaller health problem occurred, the participant's decision of going to work even if feeling ill seemed to be affected by their location; onshore or offshore? One participant explained:

*1/5: [I]f you have a cold ... if I was onshore I probably would have spent a day or two in bed, but when you know you're [already at] work then you go anyway*.

Thus, when a small health problem occurred offshore, participants made efforts to go to work, but when a similar problem arose ashore, they had the option of joining their shift after a short delay. However, a few participants indicated that they did not like being sent offshore after the shift had started because of the challenges of entering their team's established schedule. Consequently, efforts were made to start the shift at the agreed time. One participant recalled an incident exemplifying this:

*1/2: I remember a few years ago (...) a friend asked me if I had a backache and I told him yes. He said: "You need to be off sick!" "No", I answered, "I have one goal now and that's to recover so I can go offshore on Tuesday." And I did*.

### Job satisfaction

One participant claimed: *Attitudes towards work and your personality matter when you decide to go to work despite illness *(3/1). A positive attitude towards one's own work is often regarded as *job satisfaction*. The concept of job satisfaction was repeatedly used as an argument to go to work even if feeling ill:

*3/2: If you're satisfied with your place of work and kind of enjoy it, then I think it's easier to go to work and just perform, even though you feel a bit unwell. If you hated it and didn't enjoy it at all and even dreaded it, then it would be so much easier to be off sick*.

A participant demonstrated this in the following excerpt:

*1/1: On the last trip offshore, I should've stayed in a hospital bed or at least stayed at home. But I came out ... I increased my limit of endurance a bit*.

Moderator: Why would you do that?

*1/1: I like my work so much so I really wanted to come out*.

Moderator: What about your own health?

*1/1: Well, I suffered a bit. I was assigned to less demanding tasks, so I just felt that I would tolerate going to work. (...). I'm quite fortunate because I don't have a specific quantity of work to do every day. I have free rein and therefore I chose to go to work. But the main reason is that I am satisfied with my job*.

Offshore work differs from onshore work in several ways, and the participants' understanding of the concept of job satisfaction seemed to include aspects that may not be embraced by individuals working eight-hour days onshore. The participants stated that in order to cope with their physically confined working environment - the oil platform, the long working hours, as well as the constant presence of colleagues - they had to embrace the platform as a 'home away from home': *When you're out here, you don't have your family around you, so you sort of depend on your colleagues (1/5)*. They not only worked together, they *lived *together. Hence, colleagues became friends and, to some extent, even replacements for absent family. A term frequently used, to describe the emotional closeness among the workers in this environment, was 'family': *It's like we have two families: one at home and one out here ... it's almost like that (2/4)*. All focus groups voiced familiarity amongst colleagues as a necessary and positive element of offshore life. Familiarity allowed them *to involve themselves if someone else is having a bad day (...) (3/5) *and thus, either help the person concerned, or get help for them.

### Being a team worker

The CS staff work in teams, each team having designated areas to cover, including cabins and common areas, in addition to the onboard laundry service. Each team member was given a specific area, e.g. a floor. When finished with this area, the team member was expected to help other team members who still had work left. The kitchen teams worked differently in following a task rotation system.

Attending work with reduced work ability might affect colleagues' workload negatively since their help to perform certain work tasks was often required. In theory, the rule was clear: *If you're so ill that you become an inconvenience for your co-workers, then you stay off sick (2/6)*. Nevertheless, it was argued that showing up for work with reduced work ability would be better than leaving an entire workload for colleagues to handle: *You know that your colleagues will have a harder day at work if you don't go down there (3/7)*. Having a replacement sent from ashore could take days, so efforts were made to show up for work even if feeling unwell, in which case co-workers helped by easing the workload:

*2/7: If someone has a backache or something like that, we'll have them do a lighter job. We'll do the heavy work, and just wait and see if they'll get better*.

Knowing that adjustments might be provided to ease the workload was an important condition for attending work despite feeling unwell. Not all adjustments were done *for *the individual, it was expected that some adjustments were made *by *him or herself. The willingness to learn how to perform work tasks in a less demanding manner from a more experienced team member was one way of adjusting:

*2/1: As an experienced oilrig worker you've learned how to work efficiently and you can teach your less experienced colleagues how to ease their work tasks. Accessibility to equipment makes work less demanding and might allow you to manage to work despite pain and disability. However, co-operation and goodwill is needed to succeed*.

However, apparent in the discussions was the fact that *the goodwill *was limited. Participants were specific regarding restricting collegial assistance: *... you're able to go a few days, but by then we should have a replacement (2/7)*.

### Leadership and company policy

*1/5: This decision (to go to work or not) is also influenced by the culture of the work place, the expectations of my colleagues, and the company*.

The CS leaders had a central function onboard, being the link between the individual, the team, and the company. The participants' discussions on current leadership entailed respect and appreciation:

*2/4: And he is a good mixer - and only sticks his head out whenever he has to make a decision, right? Or delegate or inform or ... Otherwise we're a team, everybody. It's one big team*.

Appreciation was frequently voiced about leaders who did not hover over the individual team member, but trusted each of them to perform the agreed job. These leaders also provided equipment and resources needed by the team or the individual.

Health promotion and reducing sickness absence were major aims in present company policy and reflected a strong focus on maintaining a healthy work environment. Participants reported that they were regularly informed of the different platforms' absentee rates. Participants from a platform with high sickness absenteeism expressed discomfort towards the indirect connection made between sickness absence levels and their social work environment:

*3/5: I think it's scary to equate thriving at work with sickness absence. In comparison to other platforms, [we've] got an enormous level of sick leave, but does that say anything about the social work environment? I would like to say that this is one of the best platforms, socially speaking, that I've worked on*.

Still, participants on all three platforms commended their leaders' handling of sickness absence:

*1/2: The leaders here are really good; they'll contact people who are ill. They'll call them and talk instead of just accepting 14 days sick leave. Then it'll be two, three days and they're [back at the platform]. There is a difference between that and 14 days sick leave, because then it's suddenly six weeks before you're out again. So they're good at that. I think that's important. It's human; everybody likes the attention that shows that the boss cares*.

The leaders followed company policy when calling up the absent worker at an early stage. When possible, workers with reduced work ability were invited back to work with adjustments made to their workload or time. The leaders counselled the returning worker and arranged any necessary adjustments in the team. As a result, they were enabling a more formalized sickness presence, i.e., the individual with reduced work ability worked on terms that did not cause inconvenience for the team or jeopardize the individual's health.

## Discussion

Overall, the factors affecting the participants' decisions of whether going to work despite experiencing a health problem comprise a complex interplay of individual and work-related conditions. Main factors disclosed by the present study are the individual's own assessment of the severity of their health complaint, the location offshore, job satisfaction and familiarity, the team's ability to adjust, and the role of the CS leaders.

### Strengths and limitations of the study

Actual access to an offshore setting is one of this study's strengths. Being transported to the different platforms by helicopter enabled the researchers to conduct the focus groups in an environment familiar to the participants. There were more men than women in the groups. This is not representative for the population sample as CS staffs employ over 50% women. More female participants might have provided additional information. The reader should be attentive to the fact that participants were recruited by their CS leader. Two of the CS leaders recruited employees they saw fit to give time off from work, as the first two interviews were scheduled within normal work hours. Midway through one interview session, two participants were called back to work because of a large workload. The last interview was performed immediately after work hours. Participants in this group were also recruited by a CS leader. The possibility that CS leaders recruited participants based on their positive attitude or ability to participate in discussions must be considered. However, the CS leaders were not present during the interviews, enabling participants to partake in an open and flexible discussion. The use of groups in which participants have pre-existing personal or professional relationships has been discussed in the methodological literature on focus groups [21]. In the present study, these relationships appeared to create a supportive environment. The moderator's role was, consequently, limited to probing, rather than to managing the energy level of each participant. Still, there remains the possibility that, because of the social control and norms of the team, individual perspectives will be under-reported in such a setting [23]. A focus group discussion with colleagues is, perhaps, not well suited for discussing individual health problems. In-depth interviews could, therefore, have added more nuanced information on sickness presence from the individual's perspective.

### Sickness presenteeism in an offshore setting

In line with findings in other studies [19,26], the reasons for sickness presenteeism identified in this study can be divided into two main categories: work-related and individual. These work-related factors include time pressure caused by the shift arrangement, support by management and colleagues, own control over work situation, as well as resources available. Time pressure was never directly mentioned as a reason for going to work when ill, but the other factors were discussed. Individual factors mentioned by the participants were mainly related to ill health, job satisfaction, coping with workloads and support from co-workers and superiors, in accordance with previous findings [7,9].

The decision of whether to call in sick or not also depended on the individual's medical condition, and studies have pointed to the importance of the individual's assessment of their own health before attending work [5]. Even if feeling ill with a disease considered being minor, there was an understanding among the participants that taking a few days off could result in more rapid recovery. In a recent qualitative study from the Netherlands [27], the objective was to examine what made men and women with a musculoskeletal complaint decide whether they were too ill to work. The employees reported feeling unsure and found it difficult to judge whether absenteeism was justified. They decided either to 'play it safe'; and call in sick to avoid aggravating their health complaint, or they would seek advice from medical professionals. 'Playing it safe' was not mentioned in our material. Instead, participants described situations where they pushed themselves to attend work. Only if unquestionably ill, like having the flu, was absenteeism an option. It should be noted that economic consideration is not usually an issue when Norwegian employees' decide to go to work when feeling unwell, as the sickness benefit system grants full coverage from the first day of sick leave.

The major discovery in this study is how specific offshore working conditions have an effect on sickness presenteeism. Dew et al. [5] argue that particular workplace settings influence presenteeism in different ways. In the most benign settings, presenteeism is described as a choice, while in the least benign settings the employees feel pressured to go to work despite feeling ill. The most apparent factor in our study is the workers location when making the decision. Findings suggest that, if offshore, the individual will make an effort to attend work, as well as to be present at the start of a new shift, despite feeling unwell. The incentive for this may be found in the other factors presented by the participants: relation-related factors. A particularly interesting finding is the participants' broad understanding of job satisfaction. Location is a key element in this broad understanding, since the confinement of the platform and the long-term absence from family seems to generate a 'family relationship' among colleagues. The feeling of familiarity implies an emphasis on the meaning of teamwork and caring for colleagues, and this may provide a powerful motivation for presenteeism [5,28]. Furthermore, since workplace and home are intertwined during the shift, colleagues, leaders, and other staff can observe illness behaviour. Grinyer and Singleton [29] underscore the influence of teamwork and pressure from colleagues on decisions to turn up ill to work. The participants in our study agreed that they felt a responsibility for their team, and that this responsibility influenced the decision to turn up ill to work, but only to a certain degree. Knowing this, the participants' efforts to attend work despite illness, when already 'at work', can be interpreted in two different ways: (i) As a strategy to avoid being considered a malingerer. If actual reduced work ability due to illness is observed by the team their disapproval of a decision to be absent is likely to be minor; (ii) Feeling of team loyalty. The latter is in accordance with a study of sickness absenteeism under similar conditions of crew aboard a passenger ship [28].

Company policy and workplace settings may play important roles when employees make decisions regarding calling in sick [5]. An offshore company experienced that support from co-workers and supervisors facilitated a better work environment which in turn led to a decline in sick leave [30], suggesting that attitudes among superiors and fellow workers may prevent absence [20]. The employee's perception of organizational justice is another factor that may play a key role in protecting and enhancing employee well-being [31] and presenteeism. Lawson and co-workers [31] found that work-based social support and job demands were closely linked to both psychological health and job satisfaction. An individual's beliefs and expectations may also influence their decision regarding turning up to work ill. If the individual has experienced that going to work when ill did not have a negative health effect, this may lead to a positive expectation and accordingly influence the decision. This is in accordance with the Cognitive Activation Theory of Stress (CATS) [32].

It is evident that some of the factors identified here results from, and are even strengthened by, specific offshore working conditions. However, apart from the importance of location, the transferability of factors to other occupational groups is demonstrated in other studies. For example, the positive presence factors highlighted in Kristensen's study on Danish slaughterhouse workers [33] and in Dew and collaborators' study on hospital workers [5] are in line with our findings. This leaves research that has focused solely on the negative factors leading to sickness presenteeism largely unsupported [7,9,34].

## Conclusions

This study offers the offshore employees' perspective on the complexity of decisions regarding sickness presenteeism. Our findings show that offshore working conditions may promote sickness presenteeism. The participants' experience of a healthy and supportive work environment contributed to this outcome.

## Competing interests

The authors declare that they have no competing interests.

## Authors' contributions

KK conceived of the study and developed its methodology. KK took part in the collection of data. KK and LHM analyzed the data. Both authors drafted the manuscript, reviewed and edited drafts of the manuscript. All authors read and approved the final manuscript.

## References

[B1] Amell T Kumar S Work-related musculoskeletal disorders: design as a prevention strategy. A review J Occup Rehabil 2001 11 255 265 10.1023/A:1013344508217 11826726

[B2] Williams RM Westmorland M Perspectives on workplace disability management: a review of the literature Work 2002 19 87 93 12454354

[B3] Brage S Bjerkedal T Bruusgaard D Occupation-specific morbidity of musculoskeletal disease in Norway Scand J Soc Med 1997 25 50 57 9106947 10.1177/140349489702500111

[B4] Vahtera J Kivimaki M Pentti J Theorell T Effect of change in the psychosocial work environment on sickness absence: a seven year follow up of initially healthy employees J Epidemiol Community Health 2000 54 484 493 10.1136/jech.54.7.484 10846190 PMC1731704

[B5] Dew K Keefe V Small K 'Choosing' to work when sick: workplace presenteeism Soc Sci Med 2005 60 2273 2282 15748675 10.1016/j.socscimed.2004.10.022

[B6] Aronsson G Gustafsson K Dallner M Sick but yet at work. An empirical study of sickness presenteeism J Epidemiol Community Health 2000 54 502 509 10.1136/jech.54.7.502 10846192 PMC1731716

[B7] Aronsson G Gustafsson K Sickness presenteeism: prevalence, attendance-pressure factors, and an outline of a model for research J Occup Environ Med 2005 47 958 966 10.1097/01.jom.0000177219.75677.17 16155481

[B8] Aronsson G Gustafsson K Dallner M Sick but yet at work. An empirical study of sickness presenteeism J Epidemiol Community Health 2000 54 502 509 10.1136/jech.54.7.502 10846192 PMC1731716

[B9] Vingard E Lindberg P Hälsa, arbetsförhållanden, sjukfrånvaro och sjuknärvaro bland män och kvinnor födda 1945, 1955 och 1965. [Health, working conditions, sick leave and sickness attendance among men and women born in 1945, 1955 and 1965] 2000 Stockholm: Socialdepartementet The Ministry of Health and Social affairs

[B10] Voss M Floderus B Diderichsen F Physical, psychosocial, and organisational factors relative to sickness absence: a study based on Sweden Post Occup Environ Med 2001 58 178 184 10.1136/oem.58.3.178 11171931 PMC1740107

[B11] Elstad JI Vabo M Job stress, sickness absence and sickness presenteeism in Nordic elderly care Scand J Public Health 2008 36 467 474 10.1177/1403494808089557 18635730

[B12] Hemp P Presenteeism: at work--but out of it Harv Bus Rev 2004 82 49-58 155 15559575

[B13] Waddell G Aylward M Sawney P Back pain, incapacity for work, and social security benefits: An international review and analysis 2002 London: Royal Society of Medicine Press

[B14] Quick JC Camara WJ Johnson JV Sauter SL Hurrell JJ Jr Piotrkowski CS Spielberger CD Creating healthier workplaces: The American Psychological Association/National Institute of Occupational Safety and Health cooperative agreement. Introduction and historical overview J Occup Health Psychol 1997 2 3 6 10.1037/1076-8998.2.1.3 9552274

[B15] Karasek R Theorell T Healthy work: stress, productivity, and the reconstruction of working life 1990 New York: Basic Books

[B16] Krohne K Brage S How GPs in Norway conceptualise functional ability: a focus group study Br J Gen Pract 2008 58 850 855 10.3399/bjgp08X376131 19068158 PMC2593534

[B17] Sandman M Sykefravær og uførepensjonering: et inkluderende arbeidsliv [Sickness absence and disability pensions: an inclusive working life] 2000 Oslo: Statens forvaltningstjeneste

[B18] Nilsson K Hertting A Petterson IL Theorell T Pride and confidence at work: potential predictors of occupational health in a hospital setting BMC public health 2005 5 92 10.1186/1471-2458-5-92 16137331 PMC1208911

[B19] Hansen CD Andersen JH Going ill to work - What personal circumstances, attitudes and work-related factors are associated with sickness presenteeism? Soc Sci Med 2008 67 956 964 10.1016/j.socscimed.2008.05.022 18571821

[B20] Bellaby P Sick from work: the body in employment 1999 Aldershot: Ashgate

[B21] Halkier B Fokusgrupper [Focus groups] 2002 Frederiksberg: Samfundslitteratur & Roskilde Universitetsforlag

[B22] Krueger RA Casey MA Focus groups: a practical guide for applied research 2009 Los Angeles: Sage

[B23] Kitzinger J Qualitative research. Introducing focus groups BMJ 1995 311 299 302 7633241 10.1136/bmj.311.7000.299 PMC2550365

[B24] Giorgi A Giorgi A Sketch of a Psychological Phenomenological Method Phenomenology and Psychological Research 1985 Pittsburgh, Pa: Duquesne University Press 9 22

[B25] Malterud K Kvalitative metoder i medisinsk forskning - en innføring [Qualitative methods in medical research - an introduction] 2003 Oslo: Universitetsforlaget

[B26] Yamashita M Arakida M Concept analysis of presenteeism and its possible applications in Japanese occupational health [Abstract] Sangyo eiseigaku zasshi 2006 48 201 213 10.1539/sangyoeisei.48.201 17170514

[B27] Hooftman WE Westerman MJ van der Beek AJ Bongers PM van Mechelen W What makes men and women with musculoskeletal complaints decide they are too sick to work? Scand J Work Environ Health 2008 34 107 112 18470439 10.5271/sjweh.1221

[B28] Dahl E Sick leave aboard - a one-year descriptive study among crew on a passenger ship Internat Marit Health 2005 56 1 4 16532581

[B29] Grinyer A Singleton V Sickness absence as risk taking behaviour: A study of organizational and cultural factors in the public sector Health Risk Soc 2000 2 7 21 10.1080/136985700111413

[B30] Bauer MN Odijk J Nærværsarbeid i Statoil Forpleining [Return to work strategies in Statoil catering section] Tidsskr Nor Laegeforen 2004 124 2630 2632 15534639

[B31] Lawson K Noblet A Rodwell J Promoting employee wellbeing: the relevance of work characteristics and organizational justice Health Promot Int 2009 24 223 233 10.1093/heapro/dap025 19596657

[B32] Ursin H Eriksen HR Cognitive activation theory of stress, sensitization, and common health complaints Ann N Y Acad Sci 2007 1113 304 310 10.1196/annals.1391.024 17584977

[B33] Kristensen TS Sickness absence and work strain among Danish slaughterhouse workers: an analysis of absence from work regarded as coping behaviour Soc Sci Med 1991 32 15 27 10.1016/0277-9536(91)90122-S 2008617

[B34] Johansson G Lundberg I Adjustment latitude and attendance requirements as determinants of sickness absence or attendance. Empirical tests of the illness flexibility model Soc Sci Med 2004 58 1857 1868 10.1016/S0277-9536(03)00407-6 15020004

